# Prognostic significance of liver affliction in pregnancy related acute kidney injury in an Egyptian cohort

**DOI:** 10.1038/s41598-025-19485-7

**Published:** 2025-09-18

**Authors:** Rasha Shemies, Eman Nagy, Dalia Younis, Tamer Gaber, Fatma Abozeid

**Affiliations:** 1 Mansoura Nephrology and Dialysis Unit, Mansoura University, Mansoura, Egypt; 2 Gastroenterology and Hepatology Unit, Mansoura Specialized MedicalHospital, Mansoura University, Mansoura, Egypt

**Keywords:** Acute kidney injury, Hepatotoxicity

## Abstract

Pregnancy related-acute kidney injury (Pr-AKI) is a major public health problem that poses serious maternal and fetal risks, particularly in low-resource settings. Liver affection including conditions unique to pregnancy such as Hyperemesis gravidarum, preeclampsia/HELLP syndrome, acute fatty liver of pregnancy or pre-existing liver conditions, can further compromise these adverse outcomes. Given the critical yet understudied nature of liver involvement in Pr-AKI, this research aimed to explore the pattern of liver affliction in women with Pr-AKI and its impact on maternal and fetal outcomes. This 5-year retrospective study included women with severe Pr-AKI (stage III) according to the KDIGO definition. The medical records of the included patients were reviewed for evidence of gestational liver disorders such as (HELLP syndrome, acute fatty liver of pregnancy (AFLP), intrahepatic cholestasis of pregnancy), or evidence of preexisting/coincidental liver disease. The patients’ clinical/laboratory data and outcomes were collected and analyzed. This study of 77 pregnant women with severe Pr-AKI found a high maternal mortality rate of 18.2% and fetal mortality of 39%. 35.5% of survivors in the liver affliction group developed chronic kidney disease requiring hemodialysis. Most women presented in the third trimester with preeclampsia/HELLP syndrome, other conditions included, acute fatty liver of pregnancy and preexisting liver cirrhosis. Women with liver affection presented at older gestational ages, had higher maternal mortality, more severe hematological abnormalities and worse fetal and renal outcomes. Severe liver dysfunction was the only significant predictor of maternal mortality, which was primarily observed in patients with shock liver or pre-existing decompensated liver cirrhosis. Liver affliction is not an uncommon problem among women with Pr-AKI and it is associated with higher maternal mortality and poor fetal and renal outcomes. Further research is needed to better understand the various liver diseases that arise in pregnancy and their impact on outcomes in Pr-AKI patients.

**Supplementary Information:**

The online version contains supplementary material available at 10.1038/s41598-025-19485-7.

## Introduction

Pregnancy related-acute kidney injury (Pr-AKI) is a major public health problem, characterized by an abrupt decline in kidney function occurring at any point during pregnancy and puerperium. It poses serious maternal and fetal risks, particularly in low-resource settings^1^. Over the past few decades, improved obstetric healthcare and a decline in septic abortions have likely led to a global reduction in Pr-AKI. However, high-income countries are experiencing an uptick in Pr-AKI cases. This countertrend is largely a result of better detection, and the demographic shift towards older maternal age, which often presents with more underlying comorbidities^2–4^. Pr-AKI has been associated with a diverse array of liver diseases which can significantly compromise maternal and fetal health^5^. This encompasses both gestational liver disease such as hyperemesis gravidarum (HG); intrahepatic cholestasis of pregnancy (ICP); preeclampsia (PE)/eclampsia; HELLP syndrome; and acute fatty liver of pregnancy (AFLP) as well as other acute and chronic liver disease occurring coincidentally in pregnancy^6^. The timing of Pr-AKI provides initial insights into its underlying etiology. Early in pregnancy (During the first trimester), Pr-AKI develops most often due to septic abortion or hyperemesis gravidarum, while in late pregnancy, liver affection is usually linked to preeclampsia, HELLP syndrome, other thrombotic microangiopathic syndromes, and AFLP^4^. Gestational liver disorders, in their occurrence, exhibit trimester-specific patterns, whereas non-pregnancy-specific liver diseases can occur at any time or are even diagnosed before pregnancy^7,8^.

Liver affliction in the context of Pr-AKI, whether it is unique to pregnancy or exacerbated during gestation, generally indicates a more severe disease, which is inherently associated with higher morbidity and mortality^5^. While pregnancy is uncommon in patients with chronic liver disease as most are either in a pathological anovulatory state or have passed the child-bearing age, severe Pr-AKI has been reported in this context^8^.

Various forms of liver disease may co-exist further compromising the maternal and fetal outcomes; Hepatitis C virus (HCV), a well-known independent risk factor for adverse obstetric outcomes, is associated with increased odds of Intrahepatic cholestasis of pregnancy (ICP). ICP, in turn, is associated with increased risk of pre-eclampsia; stillbirth, preterm delivery, and neonatal intensive care unit (ICU) admission^9,10^. Both HCV and ICP lead to an additive increased odd of severe maternal morbidity^10^. Preexisting liver cirrhosis is associated with an increased risk of preeclampsia, hyperemesis gravidarum, placental abruption, and postpartum hemorrhage (PPH)^11^.

Other forms of hepatobiliary diseases may occur during pregnancy. There is an increased risk of gallstones and biliary sludge even in asymptomatic pregnant women. A minority of these women may subsequently experience acute cholecystitis. Acute pancreatitis may arise in pregnancy, and both statuses are linked to the presence of biliary stones and sludge, this can further be complicated by acute kidney injury^12^.

Studies investigating the burden of liver disease in the context of Pr-AKI are scant, particularly in the developing world. Thus, this study aimed to explore the pattern of liver affliction in women with Pr-AKI and its impact on the maternal and fetal outcome.

## Methodology

This retrospective study included patients with severe Pr-AKI who were either admitted to or referred to our nephrology unit between January 2017 till December 2022. Patients’ records from 2017 to 2022 were revised and all patients who presented with stage III Pr-AKI according to the KDIGO definition throughout this period were included. Patients with stage I, II Pr-AKI admitted to the obstetric department/ICU or those with incomplete data were excluded from the study.

Sociodemographic, clinical, and laboratory data for all patients were recorded, primarily reporting liver and kidney function tests, complete blood count, coagulation profile, and other virological and serological markers performed upon clinical indication. In addition, findings on imaging studies such as abdominal ultrasound were reported. The medical records of the included patients were inspected for Hyperemesis gravidarum, preeclampsia, HELLP syndrome, AFLP, intrahepatic cholestasis of pregnancy, or evidence of underlying acute/chronic liver or hepatobiliary disease. The Ethical Committee of the Faculty of Medicine of Mansoura University approved the study and the patients to participate by the Institution Research Board (IRB) of Mansoura Medical College (R.25.03.3110).

### Definitions

Pr-AKI is defined according to the KDIGO standard definition of acute kidney injury (AKI)^13^.

HELLP syndrome: The syndrome of hemolysis, elevated liver enzymes, and low platelet count occurring during pregnancy or puerperium^14^.

Acute fatty liver of pregnancy (AFLP): Acute liver dysfunction due to fatty infiltration of liver parenchyma, which can precipitate coagulopathy, electrolyte imbalance, and multi-organ failure almost late in pregnancy^6^.

Preeclampsia^14^: Preeclampsia is defined by hypertension with SBP > or = 140 mmHg and/or DBP > or = 90 mmHg accompanied by one or more of the following new-onset conditions at or after 20 weeks’ gestation:

1. Proteinuria—Protein creatinine ratio > or = 30 mg/mmol (0.3 mg/mg) OR 300 mg or more per 24-h urine collection (or this amount extrapolated from a timed collection).
2. Other maternal organ dysfunction, including Acute kidney injury (AKI) (creatinine > or = 90mmol/l; 1 mg/dl).
  Liver involvement (elevated transaminases, e.g. ALT or AST > 40 IU/l) with or without right upper quadrant or epigastric abdominal pain)
  Neurological complications (examples include eclampsia, altered mental status, blindness, stroke, clonus, severe headaches, persistent visual scotomata).
  Hematological complications (thrombocytopenia − platelet count below 150,000/ml, disseminated intravascular coagulation (DIC), hemolysis).
3. Uteroplacental dysfunction (such as fetal growth restriction, abnormal umbilical artery Doppler waveform analysis, or stillbirth).

Intrahepatic cholestasis of pregnancy (ICP): Diagnosis is based upon pruritus usually begins in the second and third trimester associated with elevated total serum bile acid levels, elevated aminotransferases, or both, and the exclusion of disease mimics^15^.

Underlying acute/chronic liver diseases: Include acute/chronic hepatitis, liver cirrhosis of various etiologies and hepatobiliary disorders including gallstones, cholecystitis and pancreatitis.

Transaminitis: Unexplained elevation of liver transaminases levels specifically ALT (alanine aminotransferase) and AST (aspartate aminotransferase) after exclusion of other apparent causes.

### Study outcomes

The primary outcome was renal recovery (evidence of clinical and laboratory recovery of kidney function at time of hospital discharge and for a maximal follow-up period of three months after diagnosis whenever available). Accordingly, patients were classified as follows:

- Fully recovered, with serum creatinine and urine output (UOP) returning to normal,
- Non-recovered with persistent rise in serum creatinine and/ or dependence on dialysis.

Other study outcomes included.

- Maternal mortality during the period of hospitalization.
- Fetal mortality including.
- Miscarriage: defined as fetal loss occurring before 20 weeks.
- Stillbirth: defined if fetal mortality occurred after 20 gestational weeks but before birth.

Although it is a retrospective study, sample size has been considered to assess the feasibility of the study.

Sample size was calculated as: n = Z^2^ p (1-p)/e^2^ where,

- Z = confidence level at 95% (standard value of 1.96).
- P = estimated prevalence or proportions of the projected area.
- e = range of CI.

Now substituting *p* = 22% from previous study^2^ and e = 5%, we get the value of 264.

Since we have a finite population the expected case per year is 60.

The sample size for the finite population is:

$${\text{Sn = n }} \times {\text{N}}/({\text{n}} + N - 1)$$

where N is the expected case per year = 60.

Hence the sample size is calculated to be 50.

Data analysis: Information collected from the patient’s records was entered and analyzed using SPSS 20 software. Continuous data was expressed as mean (SD), and a categorical variable as number and percent (%). Quantitative data were described using median (minimum and maximum) (interquartile range) for non-normally distributed data after testing normality using Kolmogorov–Smirnov test. All tests were 2-tailed. Nonparametric tests: Mann–Whitney U test was used to compare between 2 groups, while Kruskal–Wallis between more than 2 studied independent groups. The Spearman correlation coefficient was used to correlate between continuous non-normally distributed data. A probability value (P value) of less than 0.05 was considered statistically significant.

### Variable construction and multivariable regression analysis

To optimize statistical power while maintaining clinical relevance, a composite variable “severe liver dysfunction” was created by combining cases with either transaminitis or liver cirrhosis, as both conditions represent significant hepatic impairment with similar pathophysiological implications for maternal outcomes.

Given our sample size constraints and event frequencies, Firth penalized logistic regression was employed for all multivariable analyses using the logistf package in R. Firth regression provides finite maximum likelihood estimates and addresses small sample bias and quasi-complete separation issues that can occur with standard logistic regression in small datasets.

Model selection prioritized statistical stability over comprehensiveness. Laboratory parameters demonstrating model instability (evidenced by implausibly wide confidence intervals > 100,000, suggesting quasi-complete separation) were excluded from multivariable analysis to ensure reliable estimates. Specifically, INR was excluded due to unstable parameter estimates despite penalized regression.

Comprehensive model diagnostics included assessment of events-per-variable (EPV) ratios, model convergence assessment, and likelihood ratio tests for overall model significance. For models with EPV < 10, we applied Firth regression and interpreted results with explicit acknowledgment of potential model instability. All confidence intervals and p-values were derived using profile penalized likelihood methods. Variables with p-values ≥ 0.05 were considered non-significant, and we avoided over-interpreting marginal results.

## Results

Women included in the study had a mean age of 28 years and a median parity of 1 (1–2). Most of them presented during the 3rd trimester with a median gestational age of 32 (25.5–36) weeks. Hypertension has been revealed in 15.6% of the included sample while diabetes mellitus has not been reported in any of the included women. 4% had an underlying chronic liver disease and 6.5% gave a history of chronic kidney disease antedating pregnancy. Hematological abnormalities in the form of anemia, thrombocytopenia, and leucopenia/leukocytosis have been largely noted. Other laboratory parameters are represented in Table 1.

**Table 1:** Basic characteristics of the studied patients (*n* = 77).

Age, years	28 (23.25-33)
HTN	12 (15.6%)
CKD	5 (6.5%)
Chronic liver disease	3 (3.9%)
Gestational age, weeks	32 (25.5–36)
Gravidity	2 (2-3.25)
Parity	1 (1–2)
SBP	120 (95–150)
DBP	80 (60–90)
WBCs, 10^9^/L	12.10 (8–17)
Blood hemoglobin, g/dL	8.70 (7.20–10.30)
Platelets, 10^9^/L	133 (80–203)
Serum creatinine, mg/dL	2.20 (1.50-5)
Quantitative proteinuria, mg/day	1500 (150–3830)
Serum albumin, g/dL	2.83 ± 0.55
Total bilirubin, mg/dL	0.80 (0.73–1.68)
ALT, U/L	36 (23.5–91.5)
AST, U/L	37(22.75–96.75)
Serum uric acid, mg/dL	7.75 (5.30–9.80)
INR	1.08 (1-1.40)
Dialysis requirement	26 (33.8%)
Maternal mortality	14 (18.2%)
Renal recovery	48 (62.3%)
Fetal mortality	30 (39%)

The data are represented by mean ± SD, median (IQR), or N (%), as suitable.

**Fig. 1:**
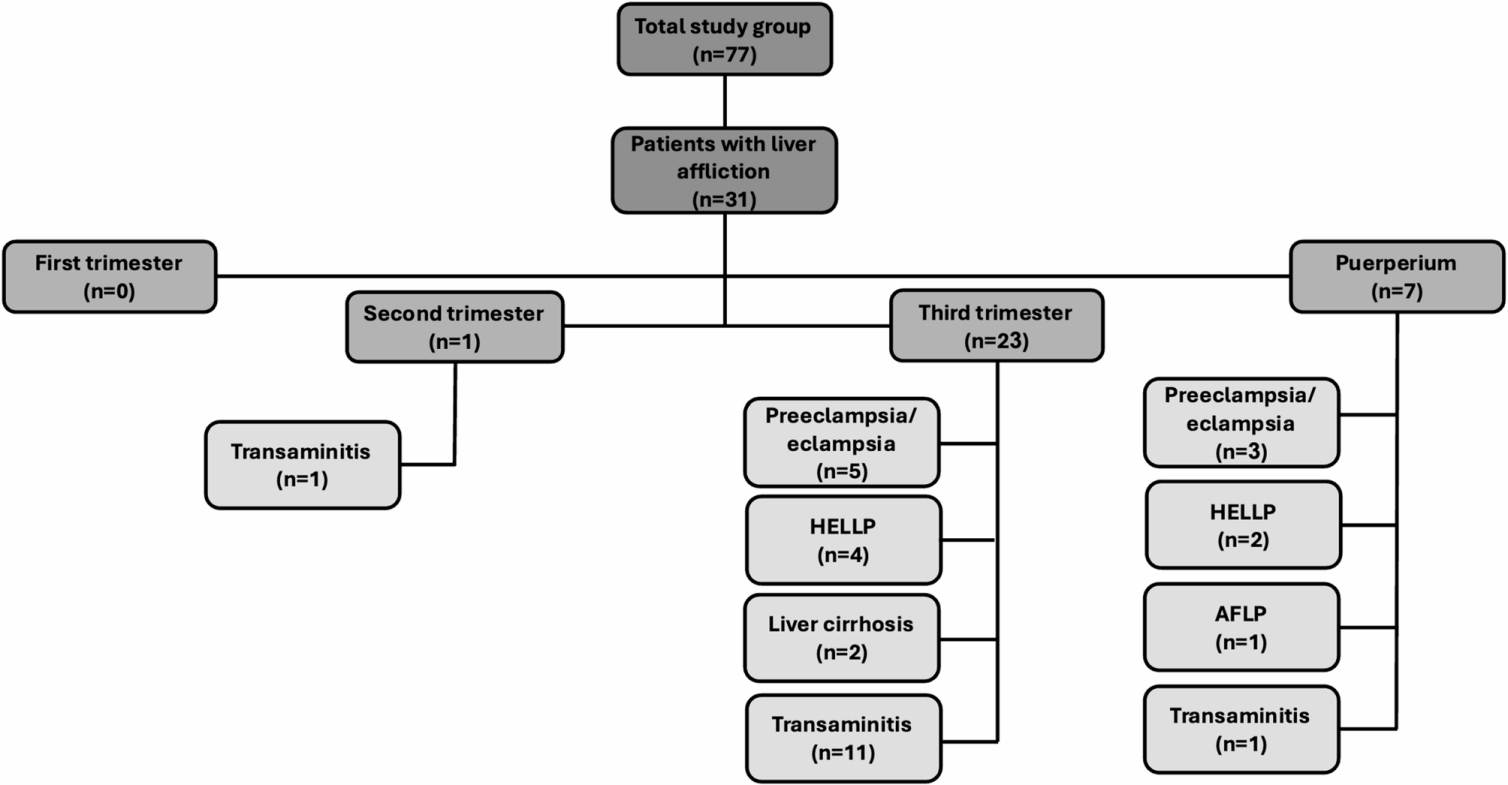
Spectrum of causes of liver injury in the studied patients according to gestational age. AFLP, Acute fatty liver of pregnancy; HELLP, Hemolysis, elevated liver enzyme levels, and low platelet levels.

The spectrum of liver affliction with Pr-AKI is displayed in Fig. 1. Most women presented during the third trimester with either preeclampsia or HELLP syndrome. A significant number (11/31) had non-specific transaminitis (likely denoting a state of shock liver). Preeclampsia/HELLP syndrome represented the main occurrence during the postpartum period. AFLP was diagnosed in only one patient immediately postpartum. None of the patients displayed any signs of liver involvement during the first trimester of pregnancy. None of the included patients had evidence of pancreatic or biliary disorders.

**Fig. 2:**
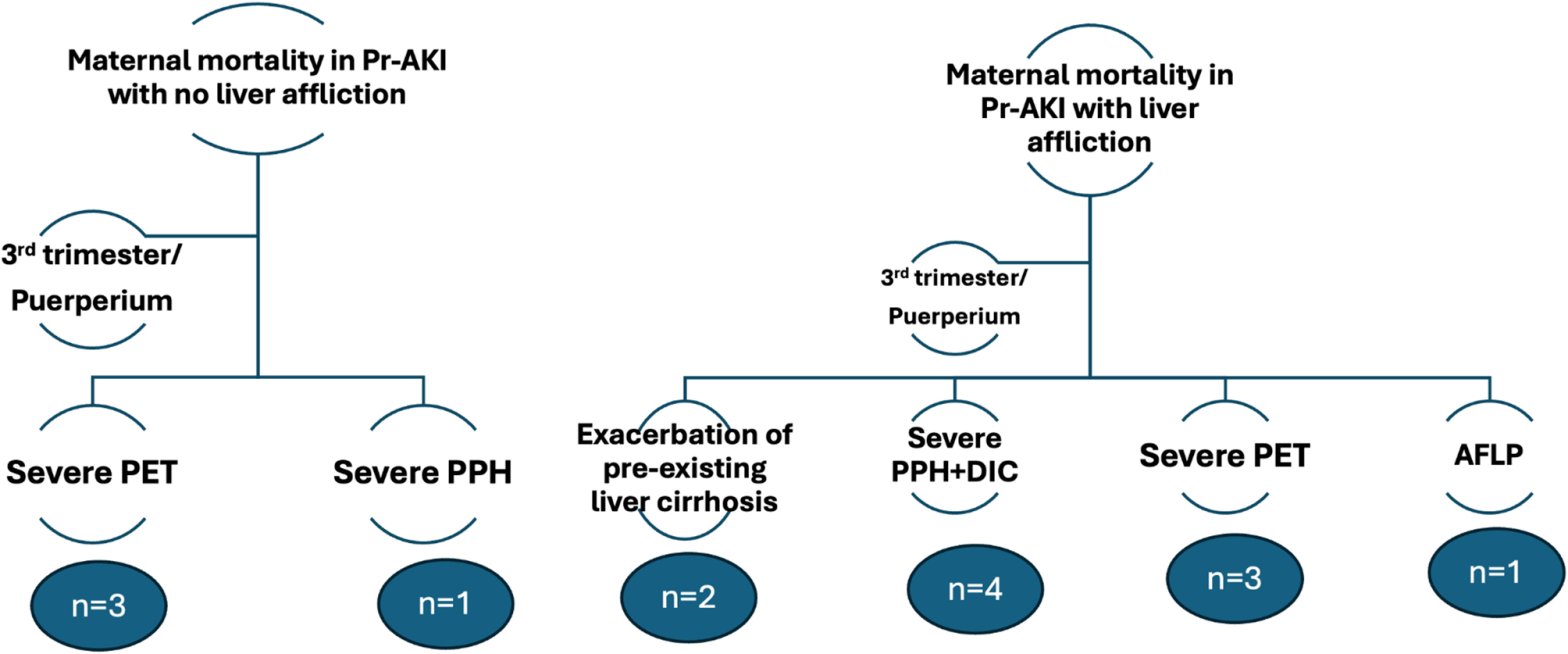
Causes of maternal mortality in patients with and without liver affliction. PET, Preeclampsia; PPH, Peripartum hemorrhage; DIC, Disseminated intravascular coagulation; AFLP, Acute fatty liver of pregnancy.

Out of the 77 women, maternal mortality occurred in 14 patients (18.2%); 4 patients had no evidence of liver affliction, and 10 patients had liver complications. In women without liver affliction, causes included severe pre-eclampsia (3 cases), and uterine atony with severe postpartum hemorrhage (1 case). For those with liver affliction, severe postpartum hemorrhage requiring massive transfusion (complicated by DIC) led to four deaths, severe pre-eclampsia to three, AFLP to one, and exacerbations of pre-existing liver cirrhosis to two. Of note, sepsis, due to secondary infections, complicated the hospital course of six women in the liver affliction group and 3 women in the other group (Fig. 2).

Among survivors, 37.66% did not recover kidney function and progressed into CKD on maintenance hemodialysis therapy. Of note, hemodialysis was the only utilized modality for the patients included. Fetal mortality was recorded at 39%.

Table 2 compared Pr-AKI patients with- versus without liver affection. It has been revealed that none of the patients with liver affection had known pre-existing CKD. They tended to present at older gestational age, which is statistically significant between the two groups. Patients with liver disease had significantly higher leukocyte count, lower platelet count and more prolonged prothrombin time. Patients with liver disease exhibited notably abnormal results of liver function tests, as anticipated. Maternal mortality was significantly higher among women with liver disease. However, fetal mortality did not significantly vary between patients with and without liver disease.

**Table 2:** Comparison between the studied patients with and without liver affliction.

	Women without liver affliction (*n* = 46)	Women with liver affliction (*n* = 31)	*P* value
Age, years	27(23–33)	29(24–34)	0.468
DM	0	0	
HTN	9(19.7%)	3(9.7%)	0.371
CKD	5(10.7%)	0	0.077
Gestational age, weeks	31(22–35)	33(28–38)	**0.046**
Gravidity	3(2–4)	2(1–3)	0.228
Parity	2(1–2)	1(1–2)	0.364
SBP	120(110–150)	130(77.5-172.5)	0.875
DBP	80(60–90)	80(47.5–100)	0.883
WBCs, 10^9^/L	11(7.45-14)	14.7(10.75–19.63)	**0.009**
Blood hemoglobin, g/dL	8.80(7.55–10.15)	8.40(6.80-10.93)	0.665
Platelets, 10^9^/L	166(107-248.5)	100(49.75–147)	**0.002**
Serum creatinine, mg/dL	2.25(1.48–5.23)	2.10(1.60–4.50)	0.905
Quantitative proteinuria, mg/day	1900(150–4000)	425(150–1875)	0.139
Serum albumin, g/dL	2.86 ± 0.54	2.79 ± 0.56	0.612
Total bilirubin, mg/dL	0.80(0.65–0.90)	1.70(0.80-6)	**0.002**
ALT, U/L	25.5(21-35.25)	143(65–418)	**< 0.001**
AST, U/L	23(21–36)	103(59.5–279)	**< 0.001**
INR	1.02(1-1.15)	1.30(1.01–1.99)	**0.001**
Serum uric acid, mg/dL	7.48(5.20–10.40)	7.80(5.40–9.40)	0.985
Dialysis requirement	15(32.6%)	11(35.5%)	0.811
Maternal mortality	4(8.7%)	10(32.3%)	**0.009**
Renal recovery	31(67.4%)	17(54.8%)	0.156
Fetal mortality	17(37%)	13(41.9%)	0.779

The data are represented by mean ± SD, median (IQR), or N (%), as suitable.

### Multivariable regression analysis

Variable construction: The composite variable “severe liver dysfunction” combined patients with transaminitis (*n* = 11) and liver cirrhosis (*n* = 3), representing 31 patients total with significant hepatic impairment.

Liver condition predictors (Tables 3, 4, 5): After applying Firth penalized logistic regression with comprehensive model diagnostics, severe liver dysfunction emerged as the only significant predictor of maternal mortality (OR 6.1, 95% CI 1.7–24, *p* = 0.006) in a model with EPV = 4.7. The overall model was statistically significant (χ^2^ = 8.17, *p* = 0.043), though the low EPV ratio necessitates cautious interpretation. No significant associations were identified between liver conditions and fetal (EPV = 10, overall *p* = 0.493) or renal outcomes (EPV = 9, overall *p* = 0.159).

**Table 3:** Link between adverse maternal outcomes in relation to the cause of liver injury.

Characteristic	Odds ratio	95% confidence interval	*P*-value
HELLP syndrome Yes	2.1	0.19, 14	0.5
Severe liver dysfunction Yes	6.1	1.7, 24	0.006
Preeclampsia/eclampsia Yes	1.5	0.14, 9.5	0.7

Firth penalized logistic regression is used to address small sample bias and separation issues. OR , Odds ratio.

**Table 4:** Link between adverse fetal outcomes in relation to the cause of liver injury.

Characteristic	Odds ratio	95% confidence interval	*P*-value
HELLP syndrome Yes	0.95	0.14, 5.4	> 0.9
Severe liver dysfunction Yes	1.3	0.43, 4.1	0.6
Preeclampsia/eclampsia Yes	0.60	0.10, 2.8	0.5

Firth penalized logistic regression is used to address small sample bias and separation issues. OR, Odds ratio.

**Table 5:** Link between adverse renal outcome in relation to the cause of liver injury.

Characteristic	Odds ratio	95% confidence interval	*P*-value
HELLP syndrome Yes	1.21	0.19, 6.17	0.8
Severe liver dysfunction Yes	2.75	0.89, 8.90	0.080
Preeclampsia/eclampsia Yes	0.84	0.14, 3.77	0.8

Firth penalized logistic regression is used to address small sample bias and separation issues. OR, Odds ratio.

Laboratory predictors (Tables 6, 7, 8): Among laboratory parameters, serum creatinine significantly predicted poor renal recovery (OR 1.3, 95% CI 1.0–1.6, *p* = 0.030) in a model with EPV = 9.0 and overall significance (χ^2^ = 46.8, *p* < 0.001). INR was excluded from analysis due to quasi-complete separation producing unstable estimates (preliminary OR > 300 with confidence intervals exceeding 800,000). Several parameters showed marginal associations (WBCs with maternal mortality, *p* = 0.057; proteinuria with fetal mortality, *p* = 0.056) but did not reach statistical significance.

**Table 6:** odds ratio of adverse maternal outcome in relation to laboratory parameters.

Characteristic	Odds ratio	95% confidence interval	*P*-value
WBCs	1.1	1.0, 1.2	0.057
Hemoglobin	1.0	0.46, 1.2	> 0.9
Serum bilirubin	1.1	0.88, 1.6	0.4
Serum uric acid	0.87	0.62, 1.2	0.4

Firth penalized logistic regression is used to address small sample bias and separation issues. OR, Odds ratio.

**Table 7:** Odds ratio of adverse fetal outcome in relation to laboratory parameters.

Characteristic	Odds ratio	95% confidence interval	*P*-value
WBCs	1.1	0.97, 1.3	0.15
Quantitative proteinuria	1.0	1.0, 1.0	0.056

Firth penalized logistic regression is used to address small sample bias and separation issues. OR, Odds ratio.

**Table 8:** Odds ratio of adverse renal outcome in relation tolaboratory data

Characteristic	Odds ratio	95% confidence interval	*P*-value
Hemoglobin	0.74	0.53, 1.0	0.067
Serum Creatinine	1.3	1.0, 1.6	0.030
Serum Bilirubin	1.1	0.98, 1.3	0.095

Firth penalized logistic regression is used to address small sample bias and separationissues. OR, Odds ratio.

Model performance and diagnostics: All models demonstrated appropriate convergence (5–13 iterations) with stable parameter estimates. EPV ratios ranged from 4.7 to 15, with Firth regression appropriately applied to all models with EPV < 10. Models with adequate EPV (Tables 4, 7) showed no significant associations, while models with lower EPV but significant findings are interpreted with appropriate caution regarding potential instability.

## Discussion

Pregnancy related acute kidney injury (Pr-AKI) is a serious event that poses an intense threat to both the mother and the fetus throughout pregnancy and postpartum. It presents a challenging definition and comprises a heterogeneous spectrum due to a multitude of underlying etiologies, with seemingly increasing incidence of Pr-AKI in the developed countries almost due to changes in the underlying maternal risk factors^1,2,16^. Complications stemming from assisted reproductive procedures, including those of ovulation induction and egg donation, have also contributed to an increased incidence of Pr-AKI in developed countries^17–20^. In the present study, most patients with Pr-AKI presented in late pregnancy or early postpartum consistent with prior research. It’s now evident that Pr-AKI is less frequently caused by septic abortions and puerperal sepsis. Instead, preeclampsia and thrombotic microangiopathies are the more common triggers, marking a clear transition in the typical onset of Pr-AKI^21–26^. Although intrahepatic cholestasis of pregnancy is generally considered the most common gestational liver disease^27^, preeclampsia/eclampsia, and HELLP syndrome were the leading causes of liver affliction in our patients in line with previous studies^26,28^. Preeclampsia/HELLP syndrome-related liver disease is a disorder unique to pregnancy, most often in the third trimester. Pregnant women with preeclampsia-induced liver disease often experience nonspecific symptoms like weight gain, right upper quadrant pain (90%), nausea/vomiting (50%), or a general viral-like illness. Jaundice occurs in 40% of cases. Severe right upper abdominal pain can be a warning sign of potential hepatic rupture^29^. The present study identified a single case of AFLP, leading to the unfortunate loss of both the mother and fetus. AFLP is a rare condition characterized by microvesicular fatty infiltration of hepatocytes, occurring in roughly 1: 7000 to 1: 16,000 pregnancies. It represents a medical and obstetric emergency that can be fatal for both the mother and the fetus unless promptly identified and managed^30^. AFLP typically begins with nonspecific symptoms: headache, fatigue, nausea/vomiting (70%), abdominal pain (50–80%), and jaundice (70%). Severe maternal complications, including hypoglycemia, acute kidney injury, and coagulopathy, affects up to 90% of patients^31^.

Notably, hyperemesis gravidarum and intrahepatic cholestasis of pregnancy were not diagnosed in any of our patients. This finding doesn’t imply their rarity in our local community, but rather indicates a more benign course not precipitating severe Pr-AKI necessitating nephrological referral.

On the other hand, our current study included three patients with pre-existing liver cirrhosis: one case was due to autoimmune hepatitis and two were due to Hepatitis C infection. In all these cases, pregnancy led to further decompensation of cirrhosis, manifesting as jaundice, portal hypertension, edema, and ascites. All the three patients also experienced preterm premature rupture of membranes (PPROM), a known risk factor for Hepatitis C (HCV) transmission to newborns. Pregnancy in women with cirrhosis is generally uncommon due to hormonal imbalances that affect fertility. Existing literature indicates that pregnancies in women with cirrhosis, particularly in advanced stages, carry a heightened risk of complications, including hyperemesis gravidarum, placental abruption, preterm delivery, and post- or intrapartum hemorrhage, as well as preeclampsia^8,11,32^.

Pr-AKI is still a major cause of maternal and fetal morbidity and mortality. Of note, the present study showed that Pr-AKI patients with liver complications presented at a later gestational age and had significantly higher maternal mortality compared to those without. The higher maternal mortality in the liver affliction group typically reflects the severity of these unique conditions that tend to present late in pregnancy, usually close to term and can rapidly progress to multi-organ failure. Unfortunately, the confounding symptoms of gestational liver diseases lead to late presentation and consequently delays in diagnosis and management that can have fatal consequences for both the mother and the fetus. In the present study, severe pre-eclampsia and obstetric hemorrhage were the primary causes of death in both comparison groups. Notably, women who died from massive peripartum hemorrhage often experienced further complications like DIC and shock liver that might explain liver affliction in those patients. The high maternal mortality rates, particularly among those referred from remote rural areas in severely compromised conditions, can be attributed to delayed referral and management. Furthermore, sepsis, arising from secondary infections, complicated the hospital stay of nine women, contributing to their mortality risk. Even in high-income settings, maternal mortality from liver disorders remains high. An analysis of U.S. death certificates shows a significant increase in maternal deaths caused by liver disorders. The rate jumped from 1.25 deaths per million live births between 1999 and 2001 to 8.80 deaths per million live births from 2016 to 2018^33,34^.

Beyond the substantial increase in maternal mortality, women with liver involvement also experienced a higher likelihood of persistent kidney dysfunction. This is likely due to the hemodynamic instability and coagulopathy associated with liver disease, which can impair kidney function and recovery^35^.

While preeclampsia/eclampsia and HELLP syndrome were the most frequent causes of liver involvement, severe liver dysfunction, presenting as either transaminitis (due to shock liver) or pre-existing decompensated liver cirrhosis was the leading cause of maternal mortality among our patients. In normal pregnancy, a physiological decrease in serum ALT is observed during the third trimester, whereas serum AST levels are typically unaffected^36^.

Transaminitis can be due to pregnancy related or non-pregnancy hepatic conditions. It might also indicate a state of shock liver (ischemic hepatitis) in patients experiencing severe obstetric hemorrhage or sepsis^30,37^. This could probably explain the association between transaminitis as an indicator of severe hepatic dysfunction and mortality in the present study. The severity of aminotransferases elevation is often used as a marker of disease severity and a predictor of adverse outcomes. Preeclampsia typically presents with elevated aminotransferase levels, where AST is initially higher than ALT. This may be due to the centrizonal pattern of liver damage and the presence of hemolysis in HELLP syndrome. While ALT levels are not markedly elevated in preeclampsia, more elevations can be found in severe preeclampsia/HELLP, particularly with hepatic infarction^27,38,39^. Hyperemesis gravidarum should remain in the differential diagnosis of severe transaminitis in the context of pregnancy, however, it typically manifests early rather than late in pregnancy. Though less common, causes such as drug-induced transaminitis, gallstone cholecystitis, and infection-related liver injury, as seen in COVID-19, have been documented^40–42^.

Accordingly, it was exceedingly difficult to confidently classify patients with isolated transaminitis under the diagnoses of preeclampsia/HELLP, AFLP, or ICP to avoid any confusion or overlapping diagnoses. Additional research is needed to fully understand the range of liver complications during pregnancy and to clarify the association between abnormal liver function tests and adverse maternal outcomes in Pr-AKI patients.

In conclusion, Pr-AKI remains a significant threat to maternal and fetal well-being, demonstrating a complex and evolving etiology. The extent of liver involvement underscores the severity of Pr-AKI. Our study shows a critical link between liver dysfunction and higher maternal mortality, as well as adverse fetal and renal outcomes. Given the high burden of liver-related maternal mortality and persistent kidney damage, further research is imperative. This research should focus on elucidating the full spectrum of liver disease in pregnancy, refining diagnostic protocols, and developing targeted interventions to improve outcomes for women with Pr-AKI and concurrent liver involvement.

### Study limitations

The study has several acknowledged limitations that might affect the generalizability and interpretation of its findings. First, it was a single center study with a relatively small patient cohort, although the study maintained adequate statistical power. Second, the retrospective nature of data collection from existing medical records introduces potential biases. Third, the study specifically included only patients with severe Pr-AKI. It is also important to note that the multivariable regression analysis utilized a composite variable “severe liver dysfunction” combining cases with either transaminitis or liver cirrhosis to optimize statistical power while maintaining clinical relevance, as both conditions represent significant hepatic impairment. These factors collectively suggest that findings should be interpreted with caution. However, we believe this study is important because it addresses an understudied area, especially in developing countries and sheds light on the liver-related complications among high-risk Pr-AKI patients, an aspect often overlooked in existing literature.

## Supplementary Information

Below is the link to the electronic supplementary material.

Supplementary Material 1

## Data Availability

Data were collected from the registered medical records following the institutional ethics committee and agreement with the Helsinki Declaration of 1975, revised in 2008.You may contact [corresponding author/profatma2000@mans.edu.eg] if someone wants to request the data from this study.
