# Supplementary material for: Prognostic significance of liver affliction in pregnancy related acute kidney injury in an Egyptian cohort
Source: Sci Rep. 2025 Sep 18;15:32603. doi: 10.1038/s41598-025-19485-7 (PMC12446491; doi:10.1038/s41598-025-19485-7)
Supplement: Supplementary file 1 — Supplementary Material 1 [file 41598_2025_19485_MOESM1_ESM.docx]

**Supplementary Table 1: Test for normality using Kolmogorov-Smirnov Z for continuous variables**

|  | Kolmogorov-Smirnov Z | P value |
| --- | --- | --- |
| Age, years | 0.145 | <0.001 |
| Gestational age, weeks | 0.145 | 0.005 |
| Gravidity | 0.207 | <0.001 |
| Parity | 0.190 | <0.001 |
| SBP | 0.074 | 0.200 |
| DBP | 0.142 | <0.001 |
| WBCs, 10^9^/L | 0.154 | <0.001 |
| Blood hemoglobin, g/dL | 0.215 | <0.001 |
| Platelets, 10^9^/L | 0.141 | <0.001 |
| Serum creatinine, mg/dL | 0.199 | <0.001 |
| Quantitative proteinuria, mg/day | 0.183 | 0.003 |
| Serum albumin, g/dL | 0.098 | 0.180 |
| Total bilirubin, mg/dL | 0.375 | <0.001 |
| ALT, U/L | 0.424 | <0.001 |
| AST, U/L | 0.367 | <0.001 |
| Serum uric acid, mg/dL | 0.147 | 0.007 |
| INR | 0.293 | <0.001 |
